# Supplementary material for: Nsp1 proteins of human coronaviruses HCoV-OC43 and SARS-CoV2 inhibit stress granule formation
Source: PLoS Pathog. 2022 Dec 19;18(12):e1011041. doi: 10.1371/journal.ppat.1011041 (PMC9810206; doi:10.1371/journal.ppat.1011041)
Supplement: S1 Fig — (A) 293A cells were infected with OC43 at MOI = 1.0 and at the indicated times post-infection mock or OC43-infected cells were treated with 500 μM sodium arsenite (As) for 50 min or left untreated (-). Whole cell lysates were analyzed by western blotting for the levels of eIF2α phosphorylation and the expression levels of the indicated host and viral proteins. (B-D) 293A cells were infected as in A and at 15 hpi mock or OC43-infected cells were treated with 500 μM sodium arsenite (+ As) for 20 or 50 min or left untreated (-). Following 50-min treatment, some cells were washed twice with PBS and provided fresh media to initiate recovery from As-induced stress for the indicated times (Wash-off). The 15 hpi time point was chosen to allow for more infected cells to form SGs for better comparison of assembly and disassembly dynamics vs. mock-infected cells. (B) Immunofluorescence analysis of SG formation in mock infected and OC43-infected cells at the indicated times post-As treatment. (C) Fraction of cells forming SGs was quantified from B. (D) Fraction of cells with SGs from C was normalized to 50 min As treatment timepoint to directly compare assembly and disassembly rates between mock and OC43-infected cells. On all graphs the two-way ANOVA and Tukey multiple comparisons tests were done to determine statistical significance (*, p -value < 0.05; ***, p-value < 0.001; ****, p -value < 0.0001; ns = non-significant). On all plots each data point represents independent biological replicate (N = 3). Error bars = standard deviation, nt = non-treated. (DOCX) [file ppat.1011041.s001.docx]

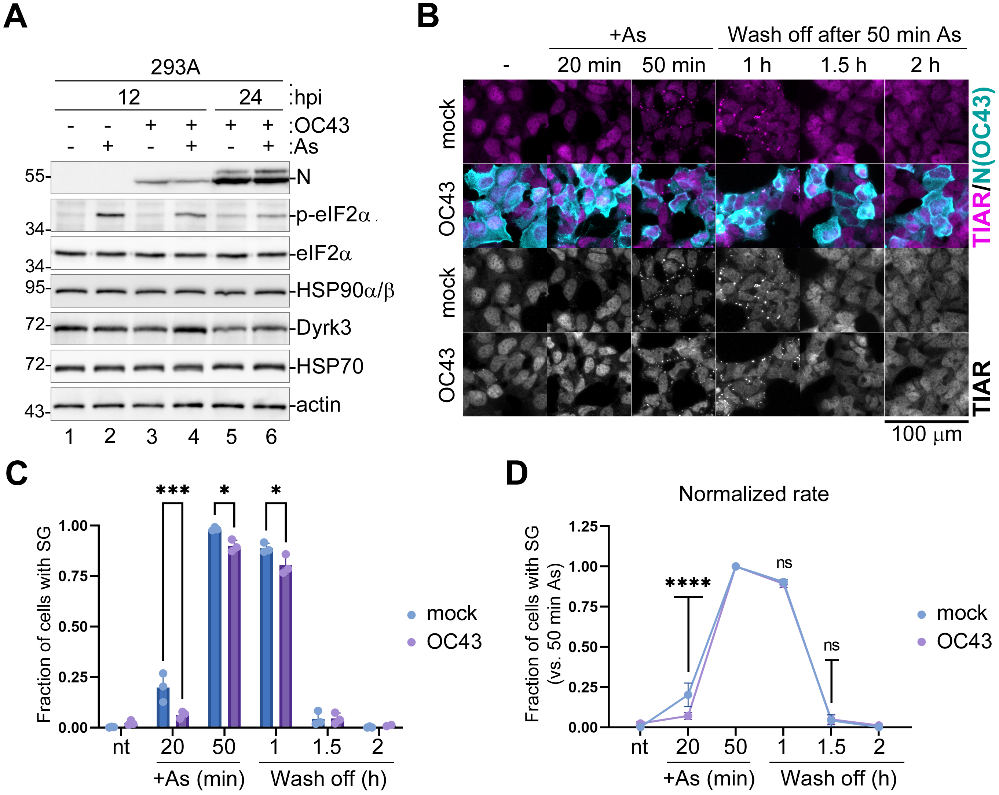


**S1 Fig. Stress granule disassembly rates are not altered in OC43-infected cells.** (A) 293A cells were infected with OC43 at MOI = 1.0 and at the indicated times post-infection mock or OC43-infected cells were treated with 500 µM sodium arsenite (As) for 50 min or left untreated (-). Whole cell lysates were analyzed by western blotting for the levels of eIF2α phosphorylation and the expression levels of the indicated host and viral proteins. (B-D) 293A cells were infected as in A and at 15 hpi mock or OC43-infected cells were treated with 500 µM sodium arsenite (+ As) for 20 or 50 min or left untreated (-). Following 50-min treatment, some cells were washed twice with PBS and provided fresh media to initiate recovery from As-induced stress for the indicated times (Wash-off). The 15 hpi time point was chosen to allow for more infected cells to form SGs for better comparison of assembly and disassembly dynamics vs. mock-infected cells. (B) Immunofluorescence analysis of SG formation in mock infected and OC43-infected cells at the indicated times post-As treatment. (C) Fraction of cells forming SGs was quantified from B. (D) Fraction of cells with SGs from C was normalized to 50 min As treatment timepoint to directly compare assembly and disassembly rates between mock and OC43-infected cells. On all graphs the two-way ANOVA and Tukey multiple comparisons tests were done to determine statistical significance (*, p -value < 0.05; ***, p-value < 0.001; ****, p -value < 0.0001; ns = non-significant). On all plots each data point represents independent biological replicate (N = 3). Error bars = standard deviation, nt = non-treated.
