# Supplementary material for: Nsp1 proteins of human coronaviruses HCoV-OC43 and SARS-CoV2 inhibit stress granule formation
Source: PLoS Pathog. 2022 Dec 19;18(12):e1011041. doi: 10.1371/journal.ppat.1011041 (PMC9810206; doi:10.1371/journal.ppat.1011041)
Supplement: S2 Fig — Expression of EGFP-tagged CoV2 Nsp1 was induced in 293A[iEGFP-CoV2-Nsp1] cells by treatment with doxycycline (+ Dox) for 24 h. (A) Western blot analysis of cytoplasmic (Cyt.), wash (Wash), and nuclear (Nuc.) fractions of induced and uninduced cells using antibodies for TIAR, cytoplasmic marker β-Tubulin, and nuclear marker Lamin A/C. (B) Nuclear TIAR band intensities from A were quantified and the normalized values to Lamin A/C were plotted (Nuc. lysate, left); ratios of nuclear to cytoplasmic TIAR band intensities were calculated from A (Nuc./Cyt., right). Unpaired Student’s t-Tests were done to determine statistical significance (*, p -value < 0.05; ns = non-significant). Each data point represents independent biological replicate (N = 3). Error bars = standard deviation. (C) Immunofluorescence analysis of induced (+ Dox) and uninduced cells, untreated or treated for 4 h with 5 μg/ml actinomycin D (+ ActD). Asterisks highlight nuclei of EGFP-Nsp1 negative cells with depleted TIAR singnal. Arrows indicate EGFP-Nsp1 positive cells with increased nuclear TIAR staining. Scale bar = 50 μm. (DOCX) [file ppat.1011041.s002.docx]

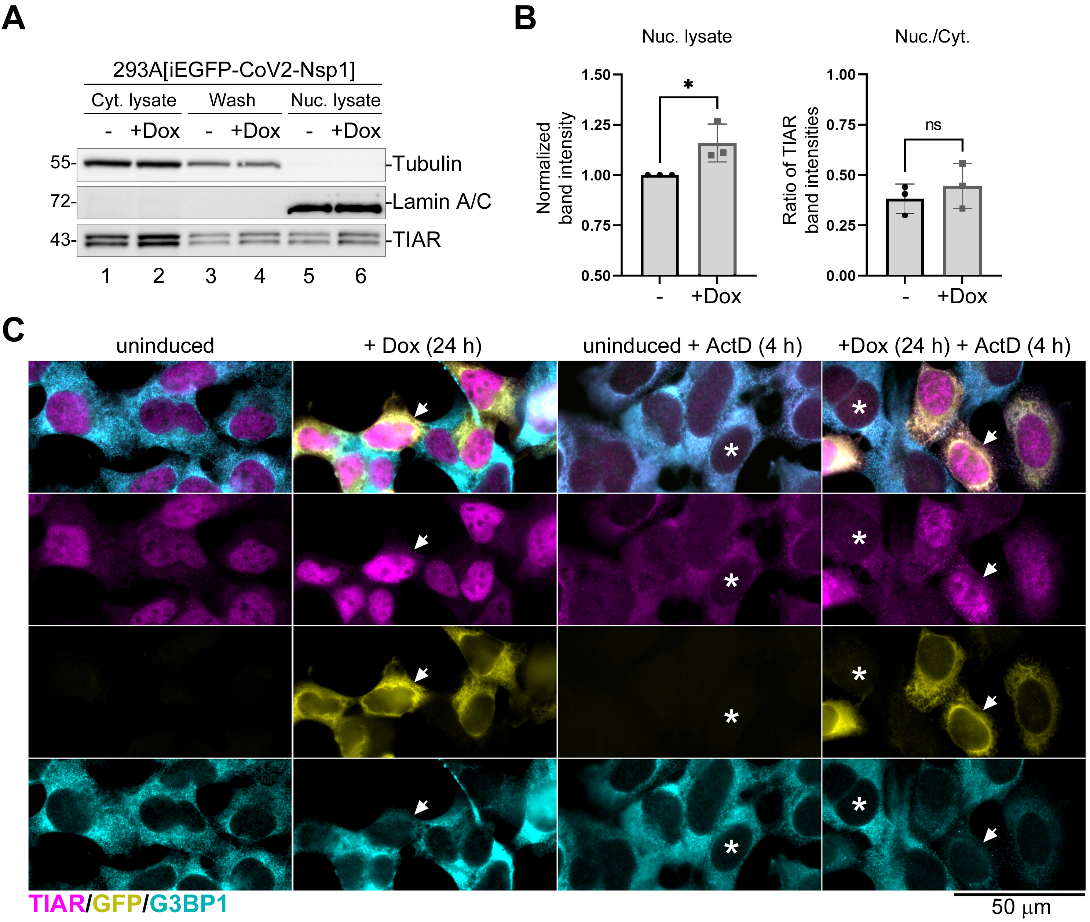


**S2 Fig. CoV2 Nsp1 increases nuclear TIAR and inhibits TIAR export following transcription inhibition.** Expression of EGFP-tagged CoV2 Nsp1 was induced in 293A[iEGFP-CoV2-Nsp1] cells by treatment with doxycycline (+ Dox) for 24 h. (A) Western blot analysis of cytoplasmic (Cyt.), wash (Wash), and nuclear (Nuc.) fractions of induced and uninduced cells using antibodies for TIAR, cytoplasmic marker β-Tubulin, and nuclear marker Lamin A/C. (B) Nuclear TIAR band intensities from A were quantified and the normalized values to Lamin A/C were plotted (Nuc. lysate, left); ratios of nuclear to cytoplasmic TIAR band intensities were calculated from A (Nuc./Cyt., right). Unpaired Student’s t-Tests were done to determine statistical significance (*, p -value < 0.05; ns = non-significant). Each data point represents independent biological replicate (N = 3). Error bars = standard deviation. (C) Immunofluorescence analysis of induced (+ Dox) and uninduced cells, untreated or treated for 4 h with 5 µg/ml actinomycin D (+ ActD). Asterisks highlight nuclei of EGFP-Nsp1 negative cells with depleted TIAR singnal. Arrows indicate EGFP-Nsp1 positive cells with increased nuclear TIAR staining. Scale bar = 50 µm.
