# Supplementary material for: Nsp1 proteins of human coronaviruses HCoV-OC43 and SARS-CoV2 inhibit stress granule formation
Source: PLoS Pathog. 2022 Dec 19;18(12):e1011041. doi: 10.1371/journal.ppat.1011041 (PMC9810206; doi:10.1371/journal.ppat.1011041)
Supplement: S3 Fig — (A) Immunofluorescence analysis of transiently transfected 293A cells expressing the indicated N-terminally HA-tagged Nsp1 constructs or EGFP control and treated with As showing recruitment of eIF3B to smaller stress granules that form in WT CoV2 Nsp1-expressing cells. (B,C) Immunofluorescence microscopy analysis for subcellular localization of the indicated protein markers in As-treated control 293A[iEGFP-CoV2-Nsp1] cells and cells treated with doxycycline (+ Dox) for 24 h to induce EGFP-Nsp1 expression. (B) Circles outline SGs in EGFP-Nsp1 positive cells that have nuclear PABP and diminished recruitment of PABP to G3BP2-positive SGs. (C) Outsets show normal recruitment of DDX3 and RNase L to EGFP-Nsp1 and G3BP1 double-positive SGs, reduced recruitment of PKR, and lack of recruitment of HSP70 and HSP90A/B in EGFP-Nsp1 expressing cells. Scale bars = 50 μm. (DOCX) [file ppat.1011041.s003.docx]

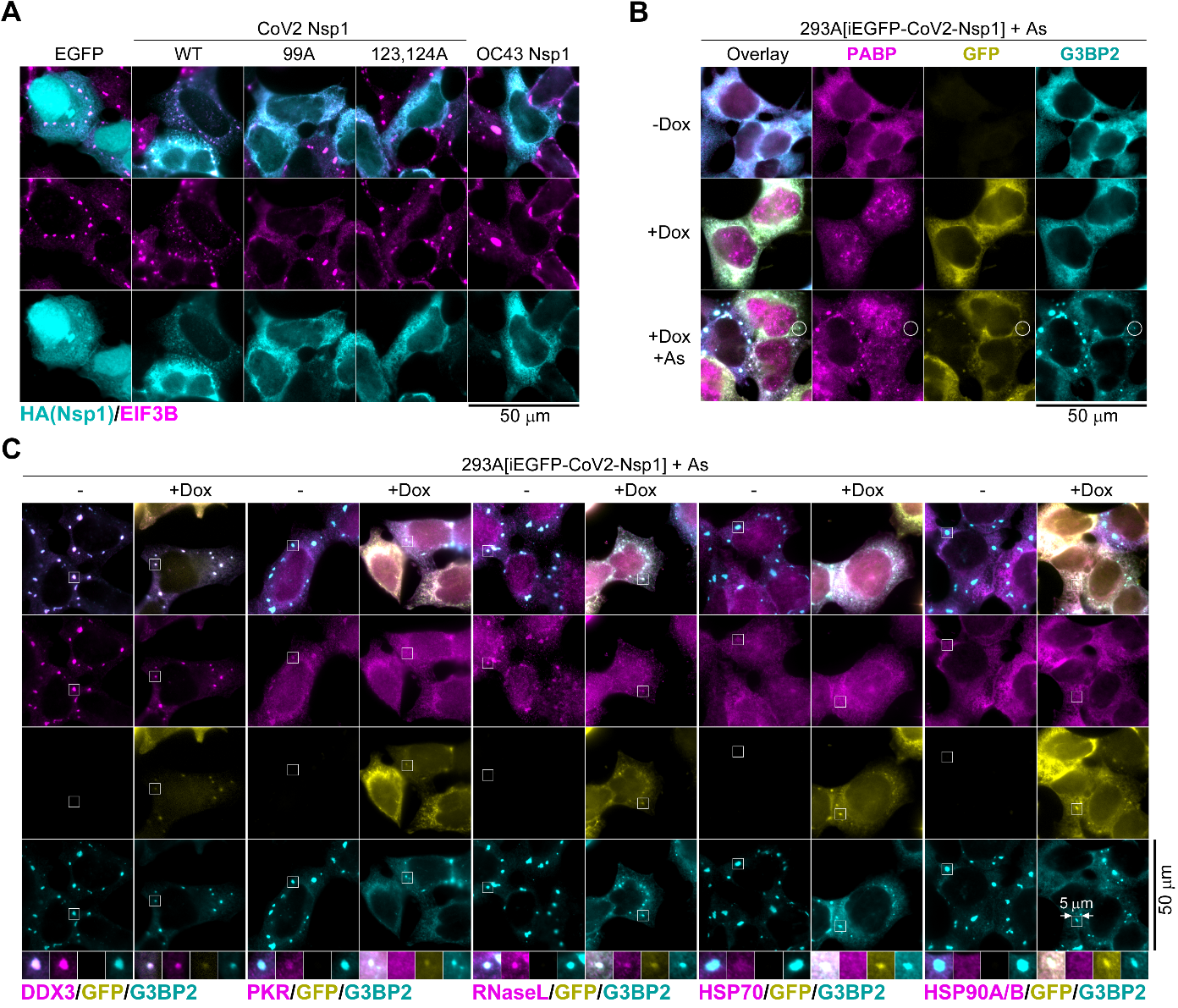


**S3 Fig. CoV2 Nsp1 expression causes nuclear relocalization of PABP.** (A) Immunofluorescence analysis of transiently transfected 293A cells expressing the indicated N-terminally HA-tagged Nsp1 constructs or EGFP control and treated with As showing recruitment of eIF3B to smaller stress granules that form in WT CoV2 Nsp1-expressing cells. (B,C) Immunofluorescence microscopy analysis for subcellular localization of the indicated protein markers in As-treated control 293A[iEGFP-CoV2-Nsp1] cells and cells treated with doxycycline (+ Dox) for 24 h to induce EGFP-Nsp1 expression. (B) Circles outline SGs in EGFP-Nsp1 positive cells that have nuclear PABP and diminished recruitment of PABP to G3BP2-positive SGs. (C) Outsets show normal recruitment of DDX3 and RNase L to EGFP-Nsp1 and G3BP1 double-positive SGs, reduced recruitment of PKR, and lack of recruitment of HSP70 and HSP90A/B in EGFP-Nsp1 expressing cells. Scale bars = 50 µm.
