# Supplementary material for: Nsp1 proteins of human coronaviruses HCoV-OC43 and SARS-CoV2 inhibit stress granule formation
Source: PLoS Pathog. 2022 Dec 19;18(12):e1011041. doi: 10.1371/journal.ppat.1011041 (PMC9810206; doi:10.1371/journal.ppat.1011041)
Supplement: S4 Fig — Immunofluorescence microscopy analysis of 293A[EGFP] and 293A[EGFP-G3BP1] cells untreated (-) or treated with arsenite (+ As) and stained for G3BP2 (magenta). GFP signal is shown in teal. Scale bar = 50 μm. (DOCX) [file ppat.1011041.s004.docx]

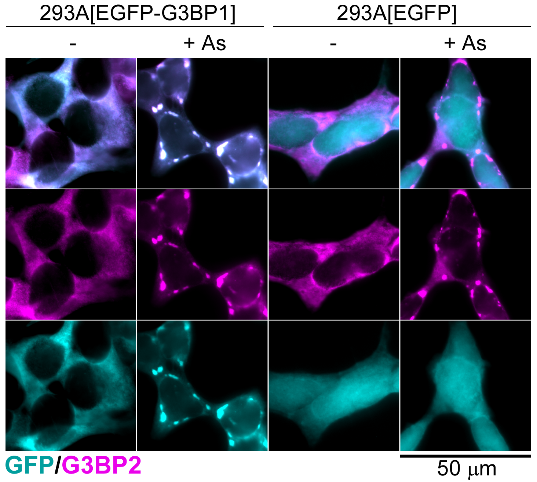


**S4 Fig. EGFP-G3BP1 overexpressing cells form As-induced SGs and do not form SGs spontaneously.** Immunofluorescence microscopy analysis of 293A[EGFP] and 293A[EGFP-G3BP1] cells untreated (-) or treated with arsenite (+ As) and stained for G3BP2 (magenta). GFP signal is shown in teal. Scale bar = 50 µm.
