# Supplementary material for: Nsp1 proteins of human coronaviruses HCoV-OC43 and SARS-CoV2 inhibit stress granule formation
Source: PLoS Pathog. 2022 Dec 19;18(12):e1011041. doi: 10.1371/journal.ppat.1011041 (PMC9810206; doi:10.1371/journal.ppat.1011041)
Supplement: Attachment — Submitted filename: Response to reviews.docx [file ppat.1011041.s005.docx]

**Response to reviews**

***Comments from the Editor:***

1. *There is a lack of data linking nsp1 function when expressed exogenously with function in the context of virus infection and this would significantly strengthen the manuscript. In addition, comparing nsp1 proteins from other CoVs, in particular those with and without mRNA degradation activity would support the conclusions made here.*

We agree that the lack of data using infection with Nsp1 mutant viruses is the limitation of our study. Nevertheless, we believe that our work provides important novel findings on the mechanisms of SG suppression by coronaviruses HCoV-OC43 and SARS-CoV2 and the functions of viral proteins N and Nsp1 in limiting SG formation. Results obtained in our viral protein overexpression studies correlate well with results from our infection model presented in this work. Generation of infectious CoV with engineered mutations requires substantial commitment of time and resources that is beyond the scope of this study. At present, we are aware of only a handful of research groups worldwide that successfully employ reverse genetic systems for generation of recombinant mutant HCoV-OC43 and SARS-CoV2 viruses. Since the time of our original submission, the analysis of Nsp1 mutant SARS-CoV2 was published by Noam Stern-Ginossar’s group, and their study confirmed the important role of Nsp1 in suppressing innate immune responses in infected cells (Fisher *et al*, 2022, *Cell Reports*). In the revised manuscript we also provide analysis of Nsp1 proteins of *Alphacoronavirus* NL63 and *Betacoronavirus* mouse hepatitis virus (MHV) that are not known to induce host mRNA degradation and the Nsp1 protein of SARS-CoV that induces host mRNA cleavage (New Fig. 7). We show that while SARS-CoV Nsp1 acts very similar to SARS-CoV2 Nsp1 in inhibiting SG formation, neither NL63 nor MHV Nsp1 proteins significantly affect eIF2α phosphorylation or SG formation in our experimental system.

2. *Information on the mechanisms behind the observations is missing. In particular, a greater characterization of the nature of G3BP2-containing SG and the antiviral mechanism of G3BP1 would be informative. Deciphering the detailed mechanism of inhibition of eIF2α phosphorylation and TIAR nuclear retention may be considered beyond the scope of the current work but should be discussed in more detail.*

In this new submission we experimentally address all the above concerns. Specifically, we examine composition of G3BP2-positive SGs that form in Nsp1-expressing cells using additional markers (new supplementary Fig. S3); we show that SG formation is increased, and viral replication and spread are decreased in G3BP1-overexpressing cells (new Fig. 8); we demonstrate that SARS-CoV2 Nsp1 protein inhibits eIF2α phosphorylation by both HRI and PERK, and that this inhibition is associated with downregulation of HRI expression and decrease of PERK activation (new Fig. 6); we provide new analyses of nucleocytoplasmic distribution of TIAR and show that SARS-CoV2 Nsp1 interferes with the export of TIAR from the nucleus in response to transcription inhibition (new supplementary Fig. S2).

3. *The role of SG disassembly in SG regulation should be included.*

We have analyzed the rates of SG assembly and disassembly in OC43-infected cells and found that the assembly of SG is affected by the virus while the rates of disassembly remain similar to mock-infected cells (New supplementary Fig. S1). We also established that OC43 infection does not upregulate the expression of SG disassembly factors HSP90a/b and Dyrk3 (Fig. S1).

4. *Quantitative data supporting the nuclear retention of TIAR should be provided.*

We conducted subcellular fractionation analyses and quantified nuclear TIAR accumulation in SARS-CoV2 Nsp1 expressing cells (new supplementary Fig. S2).

5. *A time course of SG formation in fig. 1, size and number of SG in fig. 2, SARS-CoV-2 nsp15 and use of Silvestrol in fig. 4 should be provided.*

In the new version of the manuscript, we include the time course of SG formation and eIF2α phosphorylation in the new Fig. 1 (panels 1D,E); characterization of the size and number of Silvestrol-induced SGs in the new Fig. 2 (panel 2C); analyses of the arsenite and Silvestrol-induced SG formation in both the HCoV-OC43 and SARS-CoV2 Nsp15-expressing cells in the new Fig. 4 (panels 4G-J).

6. *A more thorough discussion of existing literature is needed.*

We improved the discussion of relevant existing literature as suggested by the reviewers.

***Reviewer #1:***

*Stacia et al found that both human common cold coronavirus HCoV-OC43 and highly pathogenic SARS-CoV2 inhibit the formation of Stress granules (SGs). Mechanism study showed that both N protein and Nsp1 are responsible for this inhibition. N protein probably interferes with the formation of SGs by interaction with the SG core protein G3BP1, however, HCoV-OC43 and SARS-CoV-2 nsp1 prevent the formation of SGs by inhibition of phosphorylation of eIF2α, and SARS-CoV-2 nsp1 reduces the size and components of SGs by depletion of the mRNA level of G3BP1 and causing nuclear retention of the SG-nucleating protein TIAR. The study also confirms that G3BP1 plays anti-viral role, suggesting that SG formation may represent an important antiviral host defense that coronaviruses target to ensure efficient replication.*

*A number of previous studies have shown that Nsp1 interferes with host mRNA and ribosome, thereby inhibiting the host protein translation. As it has been demonstrated that SARS-CoV-2 Nsp1 depletes host mRNA, therefore, inhibition of the SGs formation by nsp1 is within our expectation. The novel finding is the nuclear retention of TIAR triggered by SARS-CoV-2 nsp1, and the inhibition of phosphorylation of eIF2α by both nsp1. However, the authors did not study the mechanisms of these observations. Also, the overexpression of nsp1 is not enough to demonstrate that this protein indeed inhibits SGs formation during virus infection, as in the context of virus infection, the viral protein probably co-ordinates with other viral proteins to perform its function.*

We thank Reviewer 1 for their earnest assessment of our work. We agree that the previously established activity of SARS-CoV2 Nsp1 in stimulating mRNA degradation can be hypothesized to interfere with SG condensation that is driven in part by RNA-protein and RNA-RNA interactions. Our team has previously characterized SG inhibition by the influenza A virus host shutoff endonuclease PA-X by this mechanism (Khaperskyy *et al,* 2014, *PLoS Path*). However, given that, unlike PA-X, Nsp1 is not itself a nuclease, and that the HCoV-OC43 Nsp1 was not shown to stimulate mRNA degradation, SG inhibition by Nsp1 proteins of HCoV-OC43 and SARS-CoV2 was not an obvious conclusion based on any existing literature. To our knowledge, this is the first study that convincingly show robust inhibition of eIF2α phosphorylation and SG formation by CoV Nsp1 proteins. In the revised version of the manuscript, we further analyze the mechanisms of eIF2α phosphorylation inhibition and nuclear TIAR accumulation by SARS-CoV2 Nsp1. We demonstrate that SARS-CoV2 Nsp1 protein inhibits eIF2α phosphorylation by both HRI and PERK, and that this inhibition is associated with downregulation of HRI expression and decrease of PERK activation (new Fig. 6); we provide new analyses of nucleocytoplasmic distribution of TIAR and show that SARS-CoV2 Nsp1 interferes with the export of TIAR from the nucleus in response to transcription inhibition (new supplementary Fig. S2). Finally, we agree that the lack of data using infection with Nsp1 mutant viruses is the limitation of our study. Nevertheless, results obtained in our Nsp1 overexpression studies correlate well with results from our infection model. Generation of infectious CoV with engineered mutations requires substantial commitment of time and resources that is beyond the scope of this study. At present, we are aware of only a handful of research groups worldwide that successfully employ reverse genetic systems for generation of recombinant mutant HCoV-OC43 or SARS-CoV2 viruses. Since the time of our original submission, the analysis of Nsp1 mutant SARS-CoV2 was published by Noam Stern-Ginossar’s group, and their study confirmed the important role of Nsp1 in suppressing innate immune responses in infected cells (Fisher *et al*, 2022, *Cell Reports*).

*Major Issues: Key Experiments Required for Acceptance*

*I have some major comments that the authors need to bolster their study.*

1. *This study did not investigate the mechanism of inhibition of phosphorylation of eIF2α by nsp1.*

We now provide experimental evidence that shows inhibition of eIF2α phosphorylation by SARS-CoV2 Nsp1 in response to either sodium arsenite or Thapsigargin. We demonstrate that Nsp1 affects these two stress-induced pathways differently: Nsp1 downregulates HRI kinase expression but inhibits activation of PERK without significantly affecting its expression (New Fig. 6). The detailed molecular mechanism of Nsp1 inhibition of PERK and the effects on the other two eIF2α kinases, GCN2 and PKR, warrant more focused investigation in the future, which we intend to undertake, and which is, in our opinion, beyond the scope of this study.

2. *Please check the mechanism of TIAR nuclear retention promoted by SARS-CoV-2 nsp1.*

We analysed how SARS-CoV2 Nsp1 affects nuclear export of TIAR stimulated by transcription inhibition with Actinomycin D and demonstrated that nuclear export of TIAR is inhibited (new supplementary Fig. S2). We also show that CoV2-Nsp1 may upregulate TIAR protein levels (new Fig. 6 and Fig. S2) when Nsp1 is expressed using Dox-inducible lentiviral expression vector. Very little is known about pathways involved in nucleocytoplasmic shuttling and regulation of expression of TIAR, and we believe that a more focused investigation of these processes and how they may be affected by SARS-CoV2 Nsp1 is required, which is beyond the scope of our present work.

3. *Figure 44G-4H. The author should check whether SARS-CoV-2 nsp15 inhibits SGs formation, as a parallel control experiment for Figure 4G-4H.*

We now provide analysis of SARS-CoV2 Nsp15 alongside HCoV-OC43 Nsp15 and show that these two proteins behave similarly and do not affect eIF2α phosphorylation in response to sodium arsenite in our experimental system. These two Nsp15 proteins also did not significantly inhibit SG formation in response to either sodium arsenite or Silvestrol treatment (new Fig. 4G-J).

4. *Figure 5J: How does SARS-CoV-2 nsp1 specific reduces the mRNA level of G3BP1/2 and actin, but not TIAR mRNA?*

We agree that selective resistance of TIAR mRNA to Nsp1-mediated degradation presents an interesting phenomenon. In the revised version of the manuscript, we provide focused discussion on the possible mechanisms that could be responsible for this resistance (lines 804-817). Future studies, which are beyond the scope of this work, should examine which sequence features of TIAR mRNA confer resistance to Nsp1.

5. *The reviewers’ major concerns will be addressed by investigating further the mechanism of nsp1 action, particularly what nsp1 is doing when expressed alone compared to in the context of virus, and whether SARS-CoV-2 nsp1 inhibit the formation of SGs as a consequence of reducing host mRNA levels. The authors should construct the Nsp1 defective mutant virus (R99A or R124A/K125A) to demonstrate the inhibition SGs formation by nsp1 during virus infection.*

As stated above, we agree that the lack of data using infection with Nsp1 mutant viruses is the limitation of our study. Nevertheless, we believe that our work provides important novel findings on the mechanisms of SG suppression by coronaviruses HCoV-OC43 and SARS-CoV2 and the functions of viral proteins N and Nsp1 in limiting SG formation. Results obtained in our viral protein overexpression studies correlate well with results from our infection model presented in this work. Generation of infectious CoV with engineered mutations requires substantial commitment of time and resources that is beyond the scope of this study. At present, we are aware of only a handful of research groups worldwide that successfully employ reverse genetic systems for generation of recombinant mutant HCoV-OC43 and SARS-CoV2 viruses. Since the time of our original submission, the analysis of Nsp1 mutant SARS-CoV2 was published by Noam Stern-Ginossar’s group, and their study confirmed the important role of Nsp1 in suppressing innate immune responses in infected cells (Fisher et al, 2022, Cell Reports).

6. *As both HCoV-OC43 and SARS-CoV2 nsp1 reduce the number or size of SGs, is this function conserved in coronavirus? It will be highly beneficial to compare to nsp1 from other CoVs to determine how conserved this function is. Please include more coronavirus nsp1 in this study, such as MERS-CoV, SARS-CoV, PEDV, TGEV, MHV.*

As requested by the Reviewer, in the revised manuscript we conduct analysis of Nsp1 proteins of *Alphacoronavirus* NL63 and *Betacoronavirus* mouse hepatitis virus (MHV) that are not known to induce host mRNA degradation and the Nsp1 protein of SARS-CoV that induces host mRNA cleavage (New Fig. 7). We show that while SARS-CoV Nsp1 acts very similar to SARS-CoV2 Nsp1 in inhibiting SG formation, neither NL63 nor MHV Nsp1 proteins significantly affect eIF2α phosphorylation or SG formation in our experimental system.

*Minor Issues: Editorial and Data Presentation Modifications.*

*It will also be important to include a more comprehensive discussion about existing knowledge of the function of N and nsp1 from earlier publications by others.*

We acknowledge that in the original submission, our discussion of existing literature in the Introduction could be improved. In the revised version we provide additional information and references concerning existing knowledge of the function of N and Nsp1 (lines 112-117, 127-139).

***Reviewer #2:***

*The authors studied inhibition of SG formation in HCoV-OC43-infected cells, SARS-CoV-2-infected cells, cells expressing N proteins, and those expressing nsp1 of both HCoVs. Many past studies reported that CoV replication does not induce SG formation. The authors showed that replication of HCoV-OC43 and SARS-CoV-2 did not induce the SG formation and inhibited eIF2a phosphorylation. They subsequently showed that SARS-CoV-2 replication induced the reduction of G3BP1, presumably through degradation of G3BP1 mRNA; these data represent novel discoveries.*

*The authors also examined N-mediated suppression of SG, whereas observation of N-mediated suppression of SG has been reported. So, the scientific novelty for this section is somewhat limited.*

*A notable novel discovery in this paper is that nsp1 of SARS-CoV-2 and HCoV-OC43 suppressed As-induced SG formation. SARS-CoV-2 nsp1 affected SG nucleation and composition, including nuclear accumulation of TIAR and deletion of G3BP1. The reduction of G3BP1 seems to be due to nsp1-induced degradation of G3BP1 mRNA. The authors showed nuclear accumulation of TIAR in SARS-CoV-2-infected cells and nsp1 expressing cells and suggested that nuclear accumulation of TIAR affected SG formation. Accordingly, uncovering the mechanism of nuclear accumulation of TIAR is in SARS-CoV-2-infected cells and in nsp1 expressing cells is meaningful for understanding SG formation suppression. However, the mechanism of nuclear accumulation of TIAR was not examined. Another limitation of this study is the lack of the data demonstrating the importance of nsp1-mediated suppression of SG in infected cells.*

We thank Reviewer 2 for their earnest analysis of our work. We generally agree with the Reviewer’s assessment of our novel findings and with the criticism that the lack of data using infection with Nsp1 mutant viruses is the limitation of our study. However, the results obtained in our Nsp1 overexpression studies correlate well with results from our infection model. Generation of infectious CoV with engineered mutations requires substantial commitment of time and resources that we believe is beyond the scope of this study. Since the time of our original submission, the analysis of Nsp1 mutant SARS-CoV2 was published by Noam Stern-Ginossar’s group, and their study confirmed the important role of Nsp1 in suppressing innate immune responses in infected cells (Fisher *et al*, 2022, *Cell Reports*). Based on the compelling evidence for suppression of eIF2α phosphorylation and SG formation by HCoV-OC43 and SARS-CoV2 Nsp1 proteins upon ectopic overexpression that are described in our work, future studies could be designed to directly compare efficiencies of suppression of eIF2α phosphorylation and SG formation in cells infected with the wild-type and Nsp1-mutant CoV. Alternatively, our future work aimed at identifying host factor(s) involved in the mechanism of inhibition of eIF2α phosphorylation by Nsp1 proteins should provide targets for siRNA silencing or deletion of these genes in the host cell and examining this phenotype in the wild-type virus-infected cells. Concerning nuclear accumulation of TIAR, in the revised version of the manuscript, we provide new analyses of nucleocytoplasmic distribution of TIAR and show that SARS-CoV2 Nsp1 interferes with the export of TIAR from the nucleus in response to transcription inhibition (new supplementary Fig. S2).

*Major Issues: Key Experiments Required for Acceptance*

*In the last section of the study, the authors claimed that overexpression of G3BP1 suppressed HCoV-OC43 replication. However, the data supporting this claim was limited; they only showed reduction of N-positive cells in G3BP-1 overexpressing cells at 24 h pi. Replication kinetics of HCoV-OC43 in control cells and G3BP-1-overexpressing cells should be examined and added to the paper.*

We agree with this assessment. In the revised version of the manuscript, we quantify SG formation in infected G3BP1-overexpressing cells and show that SG formation is significantly increased compared to control EGFP-expressing cells (new Fig. 8E). We also examine viral replication by analyzing infectious virus production and viral RNA accumulation, demonstrating that viral replication and spread are decreased in G3BP1-overexpressing cells (new Fig. 8F-H).

*Minor Issues: Editorial and Data Presentation Modifications.*

*The manuscript includes many grammatical errors. These should be corrected.*

We made every effort to correct any grammatical errors in the manuscript.

*Line 113-118. The authors should include a paper that shows inhibition of SG formation by 4a protein of MERS-CoV.*

As requested, we now have this reference included in the introduction (lines 124-125).

*Line 122, “43S” should be “48S”.*

In that line we referred specifically to 43S complex of the 40S small ribosomal subunit and select translation initiation factors (e.g. eIF3) as opposite to 48S preinitiation complex containing, in addition to 43S, the eFI4F complex and mRNA. While the 2015 review refers to inhibition of 48S preinitiation complex by SARS-CoV Nsp1 (Narayanan *et al*, 2015, *Virus Res.*), the latter structural study referenced in that paragraph refer specifically to 43S complex (Thoms *et al*, 2020, *Science*). We chose to use 43S specifically because of the absence of eIF4F complex on published structures.

*Line 328. Cite a reference showing inhibition of translation initiation induced by HCoV-OC43 nsp1.*

We agree with the Reviewer that this statement is not directly supported by the literature. We rephrased that sentence because we could not find a specific reference demonstrating inhibition of translation by OC43 Nsp1. Our present study shows that this protein represses EGFP reporter expression and does not induce mRNA degradation (Fig. 5), strongly suggesting that OC43 Nsp1 function is similar to functions of other CoV Nsp1 proteins that do not stimulate mRNA cleavage.

***Reviewer #3:***

*The study by Dolliver and collaborators investigate the regulation of stress granules by the coronaviruses HCoV-OC43 and SARS-CoV2 and the contribution of nsp1 proteins in this process. While several papers have reported the ability of the coronaviruses N protein to phase separate in recent months, it had remained unclear to date what the impact of human coronaviruses infection on stress granules assembly is. To address these, the authors chose to dissect how a common cold coronavirus – OC43 and the pathogenic SARS-CoV2 regulate stress granules. They report that both can impair eIF2 phosphorylation and stress granules formation and that SARS-CoV2 down-regulates the SG-scaffolding protein G3BP1. They further demonstrate using overexpression of viral proteins that nsp1 is responsible for the inhibition of eIF2 phosphorylation and that this is dependent upon nsp1-driven degradation of cellular mRNAs. They further propose that SG inhibition is a viral countermeasure to prevent the assembly of antiviral SGs given that G3BP1 overexpression alters HCoV-OC43 overexpression. Overall this study addresses a gap in our understanding of SG regulation by coronaviruses and contributes to dissecting how phase separation processes are regulated during viral infections, and the role of viral proteins in the process.* *The results and analysis are solid, well thought out, however there are a number of points that would benefit from clarification and additional work as I outline further below, and the introduction should better summarize the many studies that have dissected the role of coronavirus proteins in phase separation and regulation of SGs by coronaviruses. Previous studies using IBV (PMID: 33635931, PMID: 32422883) and MERS (PMID: 35129074, PMID: 27783669) have already dissecting how these zoonotic coronaviruses regulate SG assembly and these should be introduced. In additional several proteomics study have revealed the interaction of coronavirus proteins, including nsp1, with SG proteins or SG pathways and these should be discussed (PMID: 34774526, PMID: 32353859) especially in the context of evaluating the impact of CoVs not only on SG assembly but potentially the disassembly (see below). While the introduction underline the role of IBV nsp15 in regulating SGs, the effect of SARS-CoV2 nsp15 is only introduced later in the results section. So overall, this part would benefit from bolstering.*

We thank Reviewer 3 for their assessment of our work and describing our analysis as solid and well thought out. We also agree with the criticisms relating to the insufficient discussion of many studies on regulation of SGs by other CoVs in the Introduction section. In the revised version, we bolster our introduction and discussion sections and also specifically mention and cite work by Gerassimovich *et al,* (2021) *JBC*, and Gordon *et al*, (2020) *Nature* (lines 112-117 and 138-139) mentioned by the Reviewer. We also performed analysis of SG assembly and disassembly kinetics presented in new supplementary Fig. S1. These analyses have shown that SG assembly is inhibited in OC43 infected cells. Finally, in the revised manuscript we provide analysis of SARS-CoV2 Nsp15 alongside HCoV-OC43 Nsp15 and show that these two proteins behave similarly and do not affect eIF2α phosphorylation in response to sodium arsenite in our experimental system. These two Nsp15 proteins also did not significantly inhibit SG formation in response to either sodium arsenite or Silvestrol treatment (new Fig. 4G-J).

*Major Issues: Key Experiments Required for Acceptance*

*Figure 1: the authors demonstrate that OC43 impairs SG formation at 24hpi following arsenite stimulation and that eIF2-P is blocked in a time dependent manner. However the matching dynamic impact on SG induction is not shown and the authors should present a similar time course looking at both impairment of eIF2-P and SG induction following arsenite stimulation to evaluate whether a correlation exists between increase inhibition of eIF2-P and SG induction (showing data at 12, 24 and 48 hpi).*

These are important points and in the revised Fig. 1 we provide new analysis of the time course of inhibition of eIF2a phosphorylation and SG formation by HCoV-OC43 (Fig. 1C-E).

*Figure 2: The authors compare the ability of OC43 to block SG induction via eIF2-dependent and independent pathways and show that induction upon silvestrol is also impaired. However on panel A, when comparing OC43+Sil and mock+Sil, the SGs look bigger in mock+Sil. A thorough analysis of SG size and SG number per cell should be performed to evaluate whether OC43 infection not only impacts SG frequency but other properties.*

In this revised version we provide the analysis of SG size and number in Sivestrol-treated cells showing that in infected cell that formed SGs they were, on average, smaller and fewer (Fig. 2C).

*Figure 4: having characterised the inhibitory impact of OC43 on SG assembly the authors next evaluate the effect of N, nsp1 and nsp15 on SG assembly to assign inhibitory functions to specific viral proteins. While the results presented are very convincing, the authors should also explore whether disassembly – rather than just assembly is perturbed by CoVs. Are the defects in SG numbers due to inhibiting their assembly, or also promoting their disassembly? The proteomics study from the Krogan team (PMID: 32353859) had revealed that N also interacts not only with G3BP1/2 but with the SG-disassembly promoting factor CK2. I would suggest the authors perform recovery assay to measure how efficiently SG disassemble after stress removal (using arsenite) in the context of infected or uninfected cells. They should also measure the induction of disassembly-inducing chaperones such as Hsp90aa1 Hsp90ab1 and DYRK3 (involved in SG disassembly) are upregulated by N protein. Finally to expand on their findings that TIAR retention in the nucleus occurs, more quantitative data should be provided by performing cellular fractionation and quantifying the amount of nuclear vs cytoplasmic TIAR.*

As requested by the Reviewer, we included discussion of the potential contribution of increased SG disassembly to the SG inhibition phenotype (lines 188-191) and provided the analysis of SG assembly and disassembly kinetics presented in new supplementary Fig. S1. These analyses have shown that SG assembly is inhibited in OC43 infected cells. We also examined the levels of Hsp90ab1 and DYRK3 in OC43-infected cells and did not detect increase in their expression (new supplementary Fig. S1A).

*Figure 5: the authors show a decrease in G3BP1 level in infected cells but no impact on G3BP2 levels they then carry on assaying G3BP2 localisation showing these are smaller in nsp1 expressing cell. This raises the question whether these are canonical type I SGs or may be more akin to RNase-L bodies or other SG-like foci? Do they contain translation factors and mRNAs? Are they dependent on translation inhibition, this needs more experimental data. Given the later effect described in Fig 6 regarding the antiviral effect of G3BP1 and the previous works describing SG-localisation of several antiviral signalling components, I would also assess whether these nsp1-induced SGs contain markers previously found in SGs such as PKR, RIG-I, MDA5 for example.*

In the revised version of the manuscript, we conducted additional analyses of the composition of G3BP2-positive SGs that form in some SARS-CoV2 Nsp1-expressing cells. We show that they contain some translation initiation factors (e.g. eIF3B) and antiviral factors such as DDX3 and RNase L (new supplementary Fig. S3). Since we used overexpression for our analyses, we do not believe these G3BP2-positive granules are the RNase L granules described by Dr. Roy Parker’s group, because their formation requires dsRNA in the form of poly(I:C) and we used sodium arsenite for SG induction (Burke *et al,* (2019) *Mol Cell*).
