# Supplementary material for: Nsp1 proteins of human coronaviruses HCoV-OC43 and SARS-CoV2 inhibit stress granule formation
Source: PLoS Pathog. 2022 Dec 19;18(12):e1011041. doi: 10.1371/journal.ppat.1011041 (PMC9810206; doi:10.1371/journal.ppat.1011041)
Supplement: Attachment — Submitted filename: Response to reviews 2.docx [file ppat.1011041.s006.docx]

**Response to reviews**

***Comments from the Editor:***

*Many thanks for submission of your updated manuscript. It has now been reviewed by 2 of the previous reviewers and I am satisfied that queries from the earlier reviews have been addressed. Reviewer 1 asks for inclusion of data describing the impact of nsp1 within replicating virus. However, I agree with your assessment that generation of recombinant viruses expressing mutated nsp1 is beyond the scope of the current work and appreciate that accessing these viruses from other research groups may not be practical.*

We agree with reviewer’s assessment.

*Please see below some minor text edits for clarity:*

*1. Fig 2C - clarify that the quantitation is from sil. treated cells*

We added the annotation “+Sil.” at the top of the panel 2C.

*2. Line 620 - modify 7A to 7B.*

We corrected this typo.

*Reviewer #1: Although the editor and the reviewers pointed out that the lack of experiments examining the role of nsp1 for suppressing SG formation in infected cells represented a notable weakness of this study, the revised manuscript did not include studies using SARS-CoV-2 mutant encoding a biologically inactive nsp1. As such a SARS-CoV-2 mutant has been generated and is available to others according to ref 46, testing the role of nsp1 for suppression of SG formation in SARS-CoV-2-infected cells would be feasible without generating a new mutant SARS-CoV-2 in the authors’ lab. Inclusion of the data using mutant SARS-CoV-2 would significantly improve significance of this study.*

We agree that the lack of data using infection with Nsp1 mutant viruses is the limitation of our study. Nevertheless, we still believe that our work provides important novel findings on the mechanisms of SG suppression by coronaviruses HCoV-OC43 and SARS-CoV2 and the functions of viral proteins N and Nsp1 in limiting SG formation. Results obtained in our viral protein overexpression studies correlate well with results from our infection model presented in this work. Generation or obtaining infectious CoV with engineered mutations requires substantial commitment of time and resources that is beyond the scope of this study.

*Reviewer #1: 1. Line 386-387. The authors still stated that both OC43 and CoV2 nsp1 proteins block translation initiation. Are there any papers showing inhibition of translation initiation by OC43 nsp1? If not, this section should be altered. The data shown in Fig 5 did not prove that OC43 nsp1 suppresses translation initiation, as OC43 nsp1 may suppress another step(s) in translation or may affect protein stability.*

We agree that it has not been demonstrated that OC43 Nsp1 specifically inhibits translation initiation. Therefore, we modified that statement as follows: “…both OC43 and CoV2 Nsp1 proteins inhibit host protein expression.”

*2. Fig. 5I. Do 99A and 125A show statistically significant reduction of SG as compared with EGFP?*

We did not include this on the panel because of focus on the differences in recruitment between TIAR and G3BP2 to SGs in WT CoV2 Nsp1 expressing cells. However, the differences are statistically significant, and we now show this on the graph.

*3. Lines 461-463. This sentence should be rewritten as the meaning is not very clear.*

We have now split that sentence into two to improve clarity.

*Reviewer #2: This reviewer would like to thank the authors for provided additional results that addressed all the points raised in the previous review of their manuscript. All the points are convincingly answered and the manuscript is now worthy of publication in P.Path.*

We thank reviewer 2 for their assessment of our work and for helpful suggestions on how to improve our paper form the previous round.
